# Fabrication and characterization of novel multilayered structures by stereocomplexion of poly(D-lactic acid)/poly(L-lactic acid) and self-assembly of polyelectrolytes

**DOI:** 10.3762/bjnano.7.10

**Published:** 2016-01-21

**Authors:** Elena Dellacasa, Li Zhao, Gesheng Yang, Laura Pastorino, Gleb B Sukhorukov

**Affiliations:** 1 Department of Informatics, Bioengineering, Robotics and Systems Engineering, University of Genova, Via all’ Opera Pia 13, 16145 Genova, Italy; 2 School of Engineering and Materials Science, Queen Mary University of London, Mile End Road, E1 4NS London, UK,; 3 State Key Laboratory for Modification of Chemical Fibers and Polymer Materials, College of Material Science and Engineering, Donghua University, Shanghai 201620, P. R. China

**Keywords:** biocompatibility, layer-by-layer assembly, microcapsules, poly(lactic acids), stereocomplex

## Abstract

The enantiomers poly(D-lactic acid) (PDLA) and poly(L-lactic acid) (PLLA) were alternately adsorbed directly on calcium carbonate (CaCO_3_) templates and on poly(styrene sulfonate) (PSS) and poly(allylamine hydrochloride) (PAH) multilayer precursors in order to fabricate a novel layer-by-layer (LBL) assembly. A single layer of poly(L-lysine) (PLL) was used as a linker between the (PDLA/PLLA)*_n_* stereocomplex and the cores with and without the polymeric (PSS/PAH)*_n_*/PLL multilayer precursor (PEM). Nuclear magnetic resonance (NMR) and gel permeation chromatography (GPC) were used to characterize the chemical composition and molecular weight of poly(lactic acid) polymers. Both multilayer structures, with and without polymeric precursor, were firstly fabricated and characterized on planar supports. A quartz crystal microbalance (QCM), attenuated total reflection Fourier transform infrared spectroscopy (ATR-FTIR) and ellipsometry were used to evaluate the thickness and mass of the multilayers. Then, hollow, spherical microcapsules were obtained by the removal of the CaCO_3_ sacrificial template. The chemical composition of the obtained microcapsules was confirmed by differential scanning calorimetry (DSC) and wide X-ray diffraction (WXRD) analyses. The microcapsule morphology was evaluated by scanning electron microscopy (SEM) and transmission electron microscopy (TEM) measurements. The experimental results confirm the successful fabrication of this innovative system, and its full biocompatibility makes it worthy of further characterization as a promising drug carrier for sustained release.

## Introduction

The polycationic/polyanionic layer-by-layer (LBL) deposition on surfaces has been widely studied since the first description by Decher et al. [1–3]. The alternate adsorption of negatively and positively charged poly(styrene sulfonate) (PSS) and poly(allylamine hydrochloride) (PAH) on sacrificial templates have been the most widely characterized and applied materials for the production of hollow microcapsules [4–6]. The potential of these multilayer structures for biotechnological and biomedical applications, such as biosensors and carriers for drug delivery, led researchers to extend this technique beyond multilayer structure fabrication based on electrostatic interactions [7–11].

Over the years, other interactions such as covalent bonding [12–14], hydrogen bonding [15–17] and hydrophobic interaction [18–20] have been investigated, and also non-water-soluble polymers, viruses [21], proteins [22–26], and amphiphiles [27–29] have been used in LBL multilayers.

Among the non-water-soluble polymers, the aliphatic polyester poly(lactic acid) (PLA) has been widely used in the biomedical field due to its extraordinary biocompatibility, biodegradability and mechanical properties [19,30–33]. Lactic acid, which is the degraded product from PLA, is fully biocompatible in human bodies, and therefore medical materials made from PLA, such as surgical suture, implants, as well as drug carriers, are in high demand. Recently PLA-based polymers have been used for the fabrication of drug carriers by a LBL self-assembly technique [15,17,34]. As an example, the stepwise assembly of poly(L-lactic acid) (PLLA) and poly(D-lactic acid) (PDLA) enantiomers, forming a racemic crystal called a stereocomplex, has been successfully realized [35]. However, PLA capsules made by the LBL technique with an entirely biocompatible procedure remain a challenge [36–38].

The possibility to assemble these polymers, as well as other biocompatible polymers such as poly(methyl methacrylate) (PMMA) [39–41], poly(lactic-*co*-glycolic acid) (PLGA) [42] and poly-ε-caprolactone (PCL) [43–44], is extremely interesting for the fabrication of innovative multilayer structures to be used in drug delivery applications.

In this work, we proposed the LBL assembly of PDLA/PLLA layers onto a (PSS/PAH)*_n_*/PLL precursor (PEM) [45–46]. This innovative configuration, involving both water-soluble and non-water-soluble polymers, could represent a promising drug carrier model. The multilayer structure was first characterized on planar supports, and then transferred onto spherical sacrificial templates, in order to build hollow microcapsules. Nuclear magnetic resonance (NMR) and gas permeation chromatography (GPC) were used to characterize the chemical composition and molecular weight of synthetic PLA polymers. Ellipsometry and quartz crystal microbalance (QCM) were used to monitor the step-by-step assembly and to evaluate the thickness and the mass of the multilayers. The use of ellipsometry to characterize the layer growth gave us information about the thicknesses of the films compared to the previously used QCM technique, which only gave information about mass change [35]. Attenuated total reflection Fourier transform infrared spectroscopy (ATR-FTIR) was used to verify the stereocomplex formation and its effective adsorption onto the polyelectrolyte precursor. Differential scanning calorimetry (DSC) and wide X-ray diffraction (WXRD) analyses were also used to confirm the stereocomplex formation. The multilayer structure was then built on spherical sacrificial templates and then morphologically characterized by scanning electron microscopy (SEM) and transmission electron microscopy (TEM).

## Results and Discussion

### Chemical composition and molecular weight of PLA polymers

**Figure 1:**
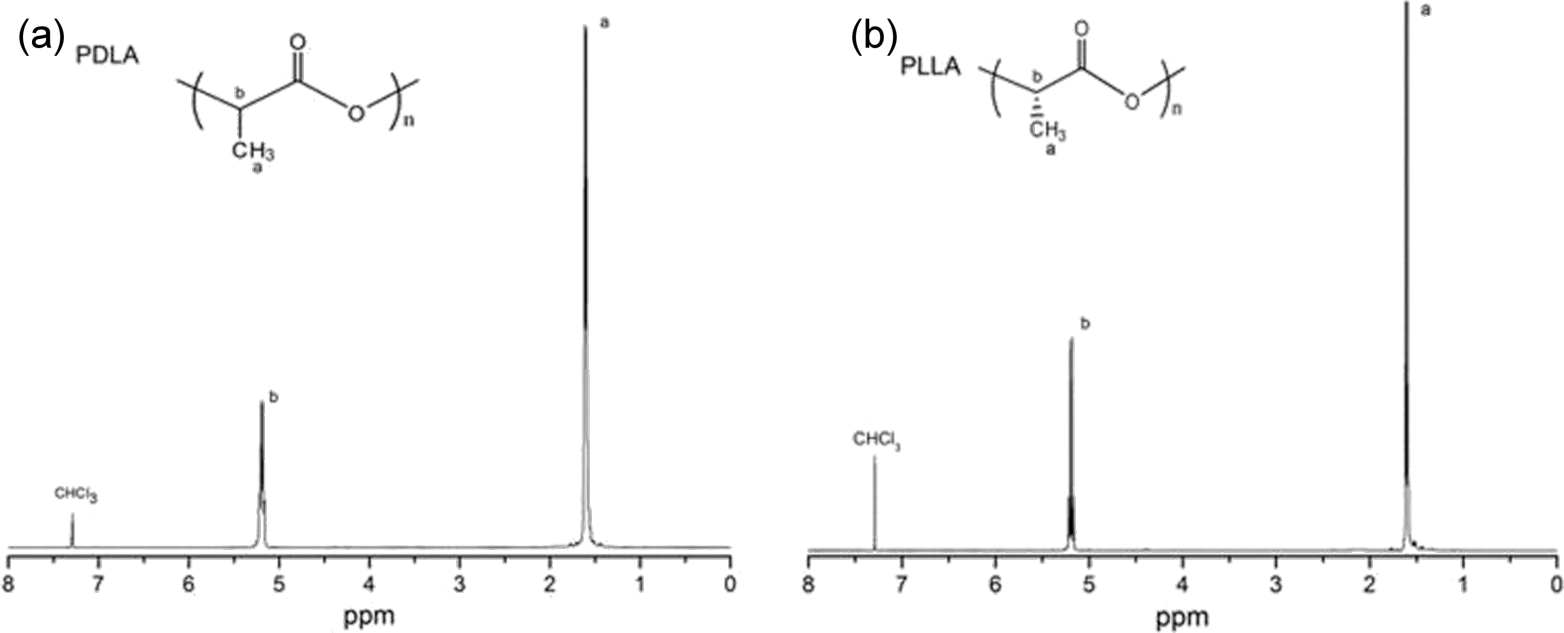
The NMR spectra for PDLA (a) and PLLA (b).

The chemical structure of PDLA and PLLA was characterized by ^1^H NMR. As can be seen in Figure 1a, the peak at 1.61 ppm belongs to the methyl group while the 5.19 ppm peak was assigned to the protons of the CH_2_ group. The small peak between 7–8 ppm was assigned to the deuterated chloroform (CDCl_3_) solvent. The spectra of Figure 1a and Figure 1b appear very similar, meaning that two polymers with the same chemical composition were synthesized.

**Figure 2:**
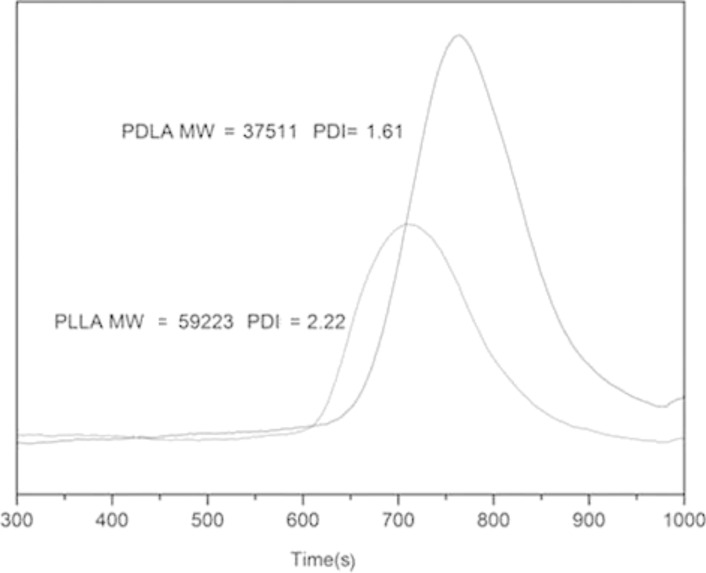
GPC curves of the synthesized PLLA and PDLA.

GPC curves shown in Figure 2 confirm that both PDLA and PLLA (having a relatively narrow molecular weight distribution) were obtained via ring-opening polymerization. The molecular weight of PDLA and PLLA are 37511 and 59223 g/mol, respectively. This was suitable for our usage due to the use of polymers with similar molecular weights in LBL assembly [46]. Thus, these polymers were used for LBL assembly directly after synthesis and purification.

### QCM measurements

**Figure 3:**
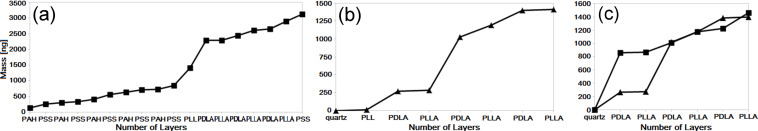
PLL/(PDLA/PLLA)_3_ multilayer deposition with (PAH/PSS)_4_/PSS precursor (a) and without (b), and (c) comparison of PLL/(PDLA/PLLA)_3_ multilayer deposition, with and without (PAH/PSS)_4_/PSS precursor.

As a first step, the LBL assembly of PEM and PLA polymers was carried out on QCM electrodes in order to monitor the effective multilayer growth. The QCM frequency shift, due to the deposition of material onto the electrode surface, was measured and the related adsorbed mass was calculated. Two kind of samples were compared, PLL/(PDLA/PLLA)_3_ multilayers deposited on (PAH/PSS)_4_/PSS multilayer precursor or directly on the crystal surface (Figure 3).

Figure 3 shows the step-by-step mass growth of the multilayer as a function of each deposited layer. Since the quartz crystal surface is mostly negatively charged, PAH was deposited as the first layer. The PEM structure shows a mean mass of 85.38 ng, with a mean frequency shift of 155.4 Hz. The total mass of adsorbed PLA layers with PEM precursor was found to be 1468 ng with a mean mass of 245 ng/layer (Figure 3a). Additionally, the total mass of the PLA layers without PEM precursor was found to be 1400 ng with a mean mass of 233 ng/layer (Figure 3b). The gradual growth of the PLA layers confirmed the successful deposition of the polymers in both cases. Comparing the two structures, no significant differences were found in terms of amount of deposited material, indicating that the PEM structure has no particular influence on the PLA adsorption (Figure 3c). However, some observations can be made about the PLA adsorption dynamics, which are better highlighted in Figure 3c. As shown in Figure 3c, in the presence of PEM precursor, the adsorbed mass of the first layer of PDLA is higher with respect to the successive layers. A similar behavior can be observed without the presence of the PEM precursor as it relates to the second PDLA layer. These differences may be due to the presence of the PEM precursor, which could have greater adsorption capability compared to the bare crystal. A higher amount of PLL adsorbed mass was also registered in the presence of the precursor (data not shown). However, no significant influence of this dynamic was observed on the final structure.

### Ellipsometry

**Figure 4:**
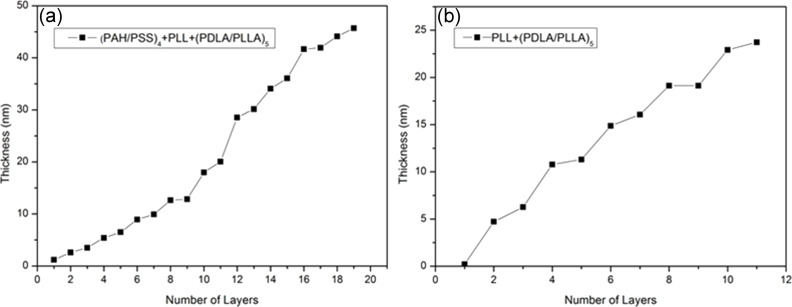
Kinetics study on the thickness of the PLL/(PDLA/PLLA)_5_ multilayer on a silicon substrate with (PAH/PSS)_4_ precursor (a) and without (b).

The kinetics of the growth of PLA films on a flat silicon substrate was also studied. Two samples were compared, a PLL/(PDLA/PLLA)_5_ multilayer deposited onto (PAH/PSS)_4_ precursor and onto bare silicon. The thickness of the PLL/(PDLA/PLLA)_5_ multilayers deposited onto the precursor as shown in Figure 4a was found to be 22.84 nm while the thickness of the PLL/(PDLA/PLLA)_5_ multilayers deposited onto bare silicon (Figure 4b) was found to be 23.5 nm, indicating again that the polyelectrolyte multilayer precursor has no particular effect on the thickness of the PLA multilayer. This is different from the results of other research on the behavior of the growth of conventional polyelectrolyte multilayers, where the underlying precursor was shown to have some influence [47].

Another phenomenon that was observed is that an odd number of PLA layers is always thicker than an even number of layers. This is due to the “dotted-structure” formation during the PLA layer deposition [35]. As the process of each PLA deposition takes longer than the polyelectrolyte assembly, each odd number layer can hardly cover the whole substrate surface uniformly within each deposition. Hence, the next even number layer deposits on the uncovered surface during the formation of the stereocomplex with the former layer, which is reflected as a thinner layer after its deposition.

### Coating of microparticles and fabrication of capsules out of stereocomplex

#### ATR-FTIR measurements

**Figure 5:**
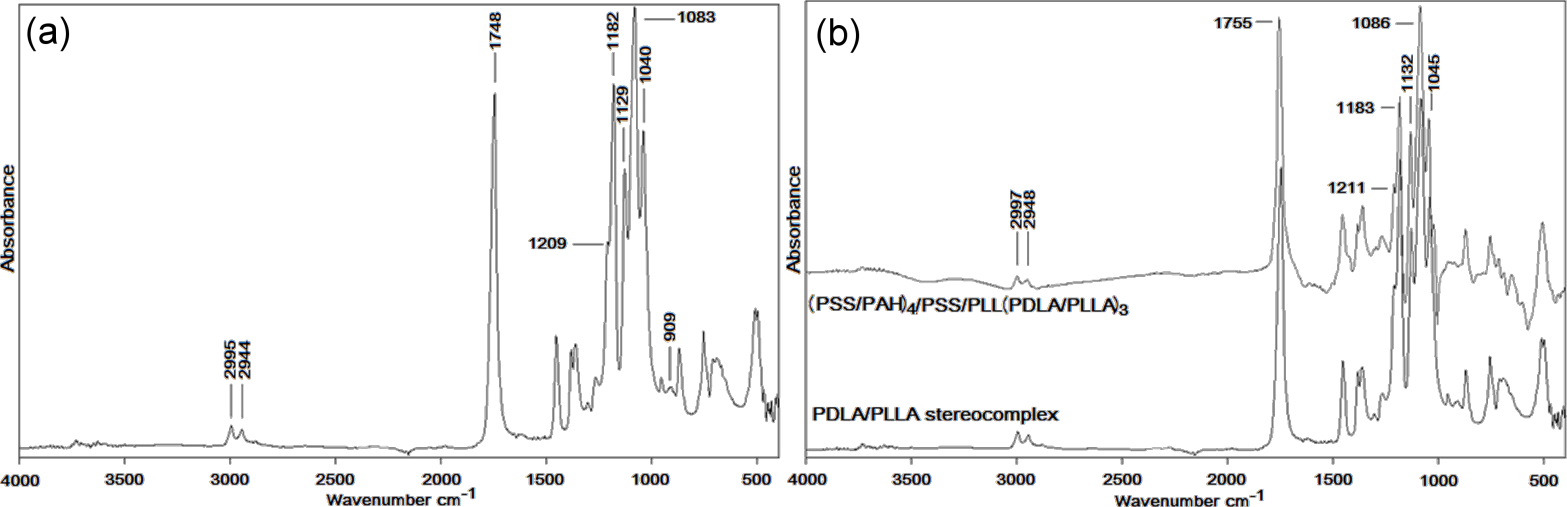
PDLA/PLLA stereocomplex spectrum by simple mixing (a) and comparison with PLA capsules with (PAH/PSS)_4_/PSS/PLL precursor (b).

The PDLA/PLLA stereocomplex formation was monitored by ATR-FTIR. Figure 5a shows the PDLA/PLLA stereocomplex spectrum obtained by mixing 1:1 solutions at 50 °C. As previously reported [48], the 1:1 blend of low molecular weight PLLA and PDLA solutions in acetonitrile is desired for the stereocomplex crystallite formation. The crystallization promotes the *v*(C=O) spectral band at 1748 cm^−1^, clearly visible in Figure 5a. Furthermore, two peaks at 909 and 1040 cm^−1^ can be identified, which are the characteristic bands of the PDLA/PLLA stereocomplex. C–O–C and C–C peaks were also visible at 1182 and 1209 cm^−1^, respectively. Finally, bands at 2995 and 2944 cm^−1^ can be assigned to the CH_3_ asymmetric stretching and CH_2_ stretching, respectively, which confirmed the successful stereocomplex formation [49–50].

Figure 5b shows the comparison between the spectra of the PDLA/PLLA stereocomplex and the capsules with PDLA/PLLA stereocomplex as outer layers. In this case, PDLA and PLLA were not mixed but rather adsorbed onto the PEM capsules by the LBL technique. The characteristic peaks of the stereocomplex were detected, confirming the successful LBL deposition of PLAs on the polyelectrolyte capsule shell.

#### WXRD curves

**Figure 6:**
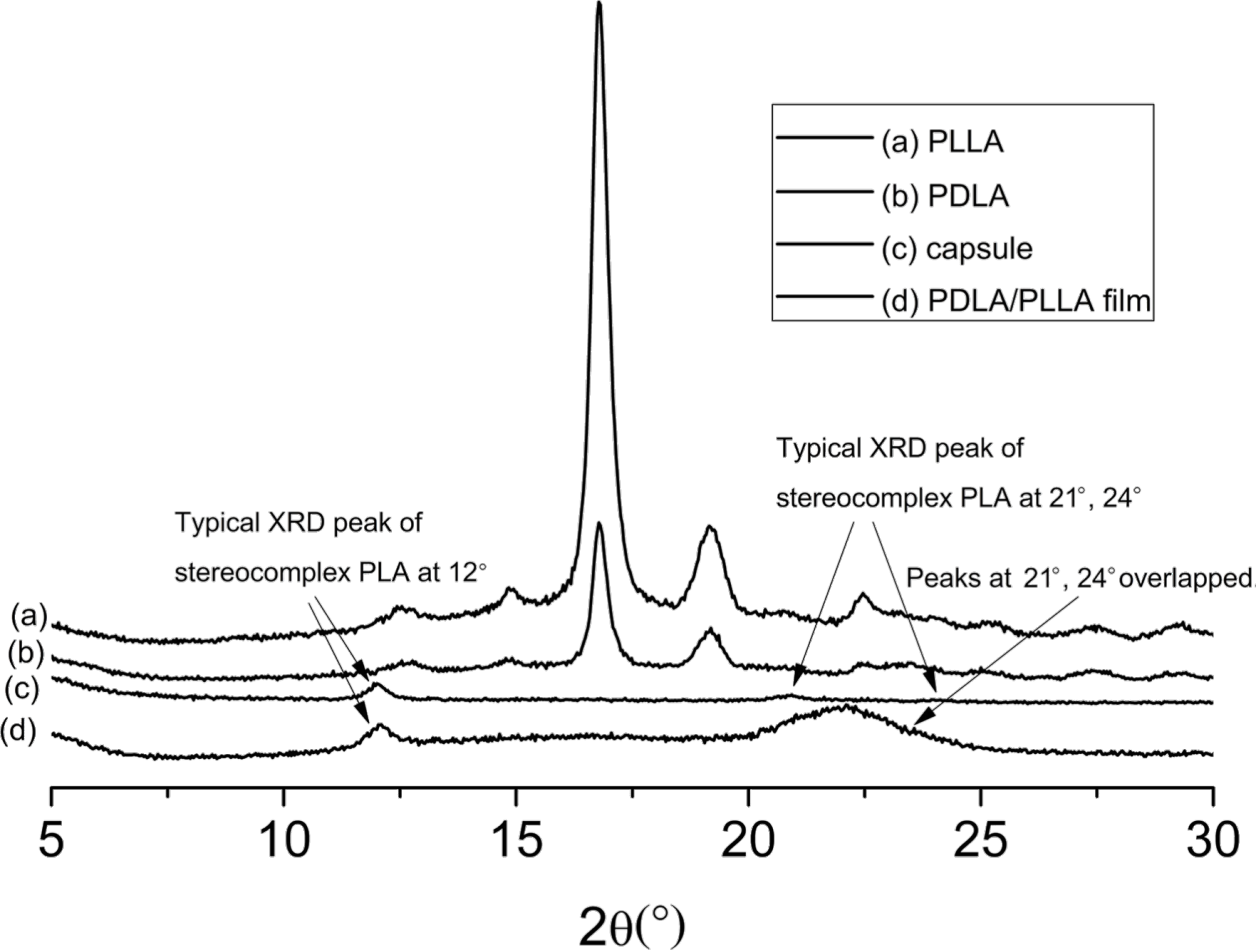
The WXRD spectra of PDLA, PLLA, PDLA/PLLA complex film and PDLA/PLLA complex microcapsules.

Since the change in crystallinity is one of the differences that occurred during the formation of the stereocomplex polymer, WXRD was used to confirm the successful formation of the PLA stereocomplex modified microcapsules [51]. It can be seen in Figure 6 that PDLA and PLLA polymers have the same diffraction peaks in the spectrum which are at θ = 15.1°, 16.5°, and 18.1° and are the typical peaks of poly(lactic acid). The diffraction peaks of the PDLA/PLLA film are at θ = 12° and 22.1° (which is an overlap of the peaks at 20.8° and 24.1°). The peaks of the microcapsules situate at θ = 12°, 20.8° and 24.1°, which are uniquely assigned to PLA stereocomplex, demonstrating that the PLA microcapsules assembled are in the structure of stereocomplex [52].

#### DSC curves

It is known that the melting point will shift to a higher degree once two enantiomeric polymers have formed their stereocomplex polymer due to the increased crystallinity. This is because the enantiomeric polymers attract each other with van der Waals force, creating a more complementary and rigid structure, which leads to a higher melting point.

**Figure 7:**
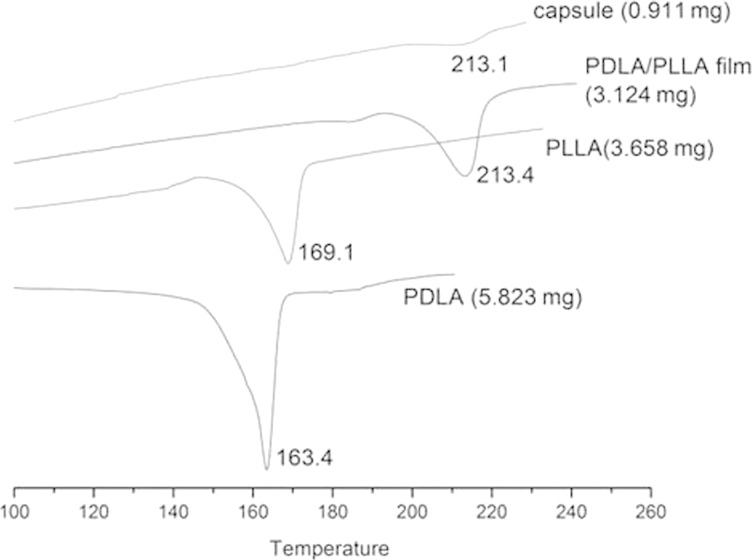
The DSC curves of PDLA, PLLA, PDLA/PLLA complex film and PDLA/PLLA complex microcapsules.

In order to know whether the PDLA/PLLA complex had been formed after the PLA microcapsules were obtained, DSC was used to measure the melting points of four different samples (Figure 7). As described in the literature, the melting points for PDLA and PLLA are approximately 170 °C, which is very close to the melting points of the PLA polymers measured in our experiment [35]. The melting points for PDLA/PLLA films and microcapsules are 213.4 °C and 213.1 °C, respectively. This result indicated that the PLA complex microcapsules had been obtained during the LBL process as the melting point of the PDLA/PLLA stereocomplex is at approximately 220 °C [35].

#### SEM measurements

**Figure 8:**
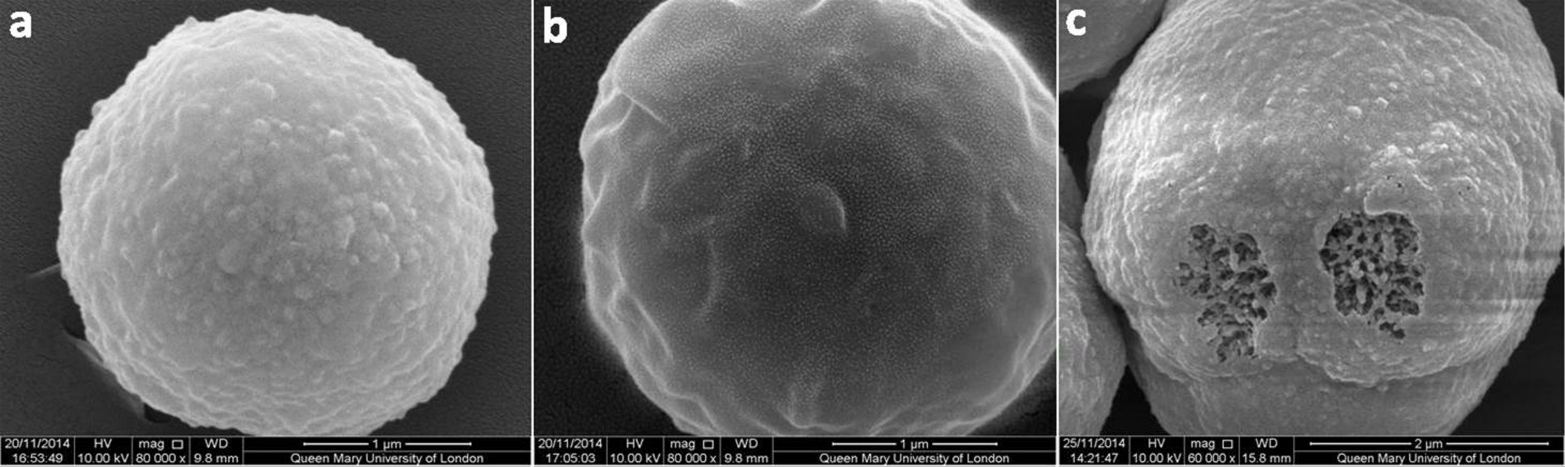
(PSS/PAH)_4_/PSS/PLL (a), (PSS/PAH)_4_/PSS/PLL(PDLA/PLLA)_3_ (b) and (PSS/PAH)_4_/PSS/PLL(PDLA/PLLA)_3_/PSS (c) multilayer structures on cores.

A qualitative characterization was also carried out by SEM measurements. Figure 8 shows two different shell structures, specifically (PSS/PAH)_4_/PSS/PLL (Figure 8a) and (PSS/PAH)_4_/PSS/PLL(PDLA/PLLA)_3_ (Figure 8b) on cores. The images clearly present different morphologies. A polyelectrolyte shell on cores, without PLA layers, shows a significant greater roughness when compared to the shell on cores with the addition of PLA. The shell with PLA layers, in fact, seems to be smoother and presents more gentle features with respect to the only PEM structure. Furthermore, from observation of Figure 8b, the shell can be considered rather homogeneous.

Finally, Figure 8c confirms the effective LBL deposition. The multilayer structure (PSS/PAH)_4_/PSS/PLL(PDLA/PLLA)_3_/PSS was built on calcium carbonate cores, and then moved from acetonitrile to water. A drop of the aqueous dispersion was let to evaporate at room temperature. In this sample, the structure of the calcium carbonate core is visible in some points where no shell is present. The different morphologies between the bare calcium carbonate core and the coated surface can be clearly noticed, confirming the successful assembly of the capsule shell. Since particles coated with (PSS/PAH)_4_/PSS/PLL(PDLA/PLLA)_3_ and (PSS/PAH)_4_/PSS/PLL(PDLA/PLLA)_3_/PSS have different terminating layers, which usually have different surface morphologies, they look different in the SEM image. As demonstrated in previous studies, particle coating layers that terminate with PLA appear smoother [53], while those with PSS as the outmost layer appear rough [54].

#### TEM measurements

**Figure 9:**
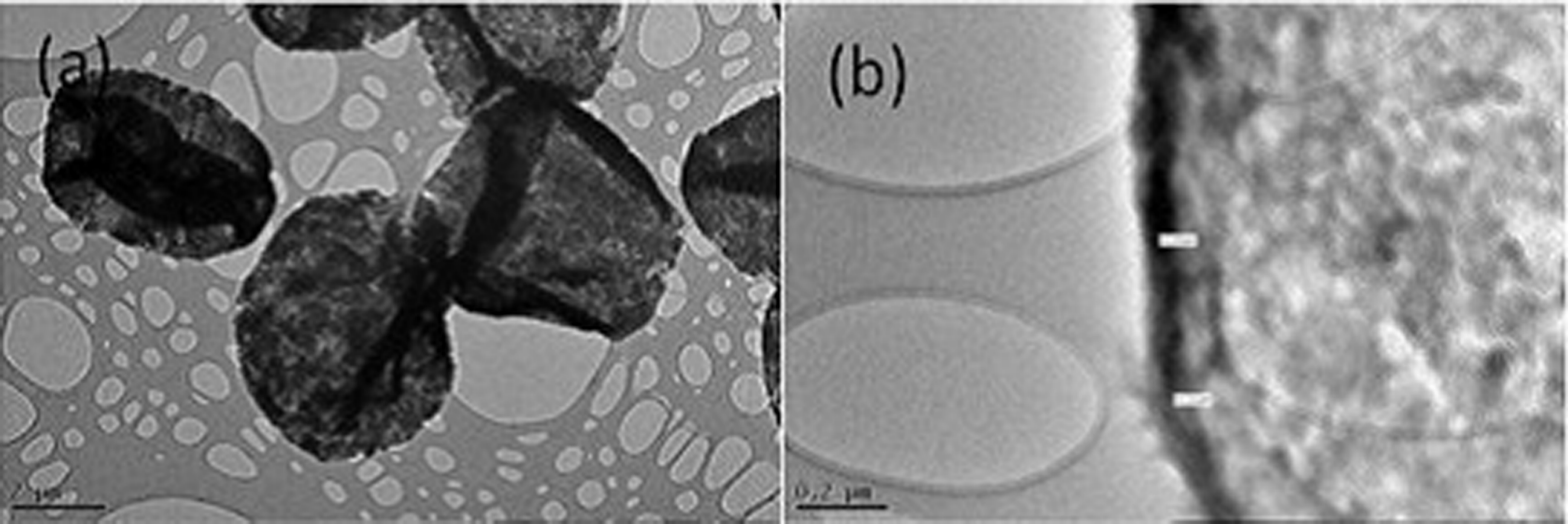
PLL(PDLA/PLLA)_10_ hollow microcapsules (a) and magnification of the PLL(PDLA/PLLA)_10_ hollow microcapsule shell (b).

Hollow microcapsules with the multilayer structure PLL(PDLA/PLLA)_10_ as the shell were characterized by TEM measurements. Figure 9a shows that most of the microcapsules look intact and flat, without evident defects, which indicates the proper core removal without damage. In addition, as can be seen in Figure 9b, the thickness of the PLL/(PDLA/PLLA)_10_ multilayer is approximately 100 nm. Hence, the thickness of each PDLA/PLLA bilayer is estimated to be 10 nm. Interestingly, the thickness of the PLA layers on the silicon substrate (see Ellipsiometry) was not as thick as that of the microcapsules. A reasonable explanation is that for the microcapsules, the layers were adsorbed on CaCO_3_ templates where the surfaces are more porous and able to accommodate more polymer within the pores of CaCO_3_. In contrast, the smooth silicon substrate is less able to adsorb polymer molecules.

pH stability of microcapsules is indeed very important as some polyelectrolytes microcapsules are sensitive to pH change. We examined our PLA microcapsules at pH 1 and 13, and no obvious change was found after 30 min of treatment with hydrochloride acid and sodium hydroxide, meaning that these PLA stereocomplex microcapsules are stable in acidic or basic conditions. This is due to the lack of pH-sensitive functional groups on the PLA polymers.

## Conclusion

PLA stereocomplex microcapsules were successfully fabricated by the LBL technique using CaCO_3_ as a sacrificial template and enantiomeric PLAs with and without PEM precursor as the shell material. This is the first attempt at fabricating PLA stereocomplex microcapsules through a fully biocompatible process.

The kinetics study and the gravimetric measurements of PLA layer adsorption on flat substrates showed a successful deposition. The presence of the PEM precursor does not seem to have a particular effect on the growth of the PLA stereocomplex layers as there was no evident difference in the thickness and mass of the PLA. The change in the melting point and crystallinity of the obtained microcapsules indicated that the stereocomplex was obtained. The presence of the stereocomplex was also confirmed by the IR measurements. The SEM images showed a qualitative difference in the template surfaces coated with PLA and PEM layers respect to those coated only with PEM precursor. The characterization by TEM confirmed a successful template removal, resulting in intact, hollow capsules.

In conclusion, a novel, multilayer structure, involving both water- and non-water-soluble polymers, was successfully fabricated. The use of the highly suitable LBL technique as a simple and inexpensive assembly technique allowed for the fabrication of stable, hollow microcapsules as promising drug delivery carriers for biomedical applications. The combination of the physical and mechanical properties of such materials could make it possible to modify characteristic features, such as surface morphology, in order to modulate important delivery factors, like permeability and release rate.

## Experimental

### Materials

Sodium carbonate, calcium chloride, poly(styrene sulfonate) (PSS, MW 70,000), poly(allylamine hydrochloride) (PAH, MW 58,000), poly(L-lysine-hydrobomide) (MW = 30,000–70,000), L-lactide, D-lactide, tannous octoate, ethylenediaminetetraacetic acid (EDTA) were obtained from Sigma-Aldrich and used without any further purification. HPCL gradient grade acetonitrile, dichloromethane and diethyl ether were purchased from Fisher Chemical and were used as received. The water used in the experiments and for the preparation of solutions was purified by a Milli-Q system and had a resistance of 18.2 MΩ·cm.

### Methods

#### Synthesis and characterization of PLLA and PDLA

Typically, 10 g of lactide and 0.5% tannous octoate (as a catalyst) were added to a conical flask. Then the conical flask was place into a vacuum oven at 180° after being sealed and the ring-opening polymerization lasted for 12 h. Afterwards, the crude polymer was dissolved in dichloromethane and precipitated in diethyl ether and this process was repeated three times to get rid of impurities and small molecules.

^1^H NMR characterization was carried out using a Bruker AV spectrometer at a frequency of 400 MHz at room temperature. CDCl_3_ and tetramethylsilane were used as solvents for the samples and internal reference, respectively. The sample concentrations were all fixed at 5 mg/mL.

The molecular weight and polydispersity index of synthesized PLAs were measured by GPC. THF was used as the eluent at a flow rate of 1.0 mL/min, while 2% of triethylamine was added to the solvent before dissolving the samples to avoid the tailing and adsorption phenomenon. The concentration of the polymer samples were all at 2 mg/mL.

#### Microcapsule preparation

Calcium carbonate microparticles (3 μm in diameter) were synthetized by mixing at 900 rpm with volumes of 0.33 M calcium chloride and 0.33 M sodium carbonate solutions according to the following reaction [55–56]:

[1]
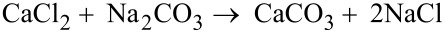


Calcium carbonate microparticles were used as sacrificial microtemplates for the assembly of polymeric microcapsules. As soon as the microparticles were synthetized, the adsorption steps (15 min in duration) of anionic PSS (2 mg/mL in 0.5 M NaCl) and cationic PAH (2 mg/ml in Milli-Q water) followed. After each adsorption step, three washings in Milli-Q water (1500 rpm for 1 min) were carried out. Once the four bilayer structures were deposited, one layer of PLL (5 mg/mL in Milli-Q water) was let to adsorb for 30 min on the top of the (PSS/PAH) multilayer, and again three washings followed. At this point, the coated templates were transferred in acetonitrile for the next PLA adsorption steps. PDLA and PLLA (5 mg/ml in 45 °C Acetonitrile) were let to adsorb for 1 h in acetonitrile at 45 °C, and each adsorption step was followed by three washing steps in acetonitrile. The process was carried out until three (PDLA/PLLA) bilayers were adsorbed, and the final structure (PSS/PAH)_4_/PSS/PLL(PDLA/PLLA)_3_ was obtained.

The same procedure was used for the fabrication of the multilayer structure PLL(PDLA/PLLA)*_n_* directly on calcium carbonate microtemplates. The obtained PLA-coated particles were then transferred back to water after washing off the organic solvent and the CaCO_3_ cores were solubilized by 0.2 M EDTA solution. The hollow microcapsules were redispersed in water and were stored in a refrigerator at 4 °C.

#### Quartz crystal microbalance

A homemade quartz crystal microbalance (QCM) with a resonance frequency of 10 MHz was used for measurements. Before the multilayer deposition, the quartz electrodes were cleaned with piranha solution (H_2_SO_4_:30% H_2_O_2_ aqueous solution, 3:1 in volume) for 2 min, followed by two washing steps in pure water and a final drying step in nitrogen flux. The amount of polymer adsorbed, Δ*m*, could be calculated by measuring the frequency decrease in the QCM, Δ*F*, using the following equation:

[2]
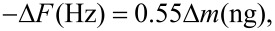


derived from Sauerbrey’s equation [57],

[3]
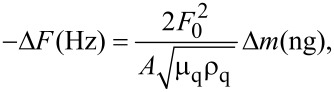


where *F*_0_ is the resonance frequency of the quartz crystal oscillator, *A* is the area of the electrode (0.205 cm^2^), ρ_q_ is the quartz density (2.648 g/cm^3^), and µ_q_ is its shear modulus (2.947·10^11^ g/cm·s^2^).

The cleaned electrodes were immersed into aqueous solutions of PSS and PAH (2 mg/mL) for 15 min and PLL (5 mg/mL) for 30 min, then taken out, rinsed thoroughly with pure water, and dried with N_2_. Since the quartz crystal surface is mostly negatively charged, PAH was deposited as the first layer.

The QCM was then immersed into acetonitrile solutions of PDLA and PLLA (5 mg/mL) for 1 h at 45 °C. Again, after removal from the PLA solutions, the coated electrodes were rinsed thoroughly with acetonitrile at 45 °C and dried with N_2_. The deposition steps were repeated until the desired multilayers, with and without PEM precursor, were obtained.

#### Ellipsometry

The thickness of the PLA films deposited on silicon substrates was measured by a J. A. Woollam alpha-SE ellipsometer. A proper model should be chosen before the measurement to fit the given substrate and to minimize the error.

Before deposition of PLA, PLL was deposited on the silicon substrate to establish an interaction with PLA to form multilayers [45]. In detail, a 1 × 1 cm silicon substrate was immersed in a polylysine solution at a concentration of 5 mg/mL at 25 °C for 30 min. After the washing and drying steps, the substrate was alternately incubated in the PDLA/PLLA solutions for a similar deposition process. The measurement of thickness was recorded after the substrate was completely dried, after each deposition step.

#### ATR-FTIR measurements

Attenuated total reflection Fourier transform infrared spectroscopy (ATR-FTIR) spectra were obtained with a Bruker A225/Q device equipped with a Bruker MCT detector. Each spectrum was recorded with a total of 32 scans at a 4 cm^−1^ resolution. The PDLA/PLLA stereocomplex was obtained by mixing 1:1, 5 mg/mL solutions at 50 °C. The samples were prepared by pouring drops of the stereocomplex and the microcapsules on glass slides. The solvent was allowed to evaporate at room temperature for at least 48 h before measurement.

#### Differential scanning calorimetry

The melting point of different PLA samples was characterized by a Mettler Toledo DSC822e instrument. The polymer powder was first filled into a steel sample holder and then was sealed by immobilizing a cap before being put into the instrument.

#### Wide X-ray diffraction

Wide angle X-ray diffraction spectrometry was employed to analyze the crystallinity of the polymers with a Siemens D5000 X-ray powder diffractometer ranging from 5° to 30°.

#### Scanning electron microscope

SEM measurements were carried out with an Inspect FEI instrument from Oxford Instruments at an operation voltage of 10 kV. A drop of the sample solution was placed onto a glass wafer, dried overnight at room temperature, and sputtered with gold before analysis.

#### Transmission electron microscope

A JEOL 2010 TEM was applied to observe the inner morphology of PLA microcapsules as well as roughly measuring the thickness of their shell. The diluted microcapsule solution was pipetted onto a copper grid and left to dry in air overnight before measurement.
